# Gridding discretization-based multiple stability switching delay search algorithm: The movement of a human being on a controlled swaying bow

**DOI:** 10.1371/journal.pone.0178950

**Published:** 2017-06-08

**Authors:** Libor Pekař, Radek Matušů, Roman Prokop

**Affiliations:** 1 Department of Automation and Control Engineering, Faculty of Applied Informatics, Tomas Bata University in Zlín, Zlín, Czech Republic; 2 Regional Research Centre CEBIA-Tech, Faculty of Applied Informatics, Tomas Bata University in Zlín, Zlín, Czech Republic; 3 Department of Mathematics, Faculty of Applied Informatics, Tomas Bata University in Zlín, Zlín, Czech Republic; Tongji University, CHINA

## Abstract

Delay represents a significant phenomenon in the dynamics of many human-related systems—including biological ones. It has i.a. a decisive impact on system stability, and the study of this influence is often mathematically demanding. This paper presents a computationally simple numerical gridding algorithm for the determination of stability margin delay values in multiple-delay linear systems. The characteristic quasi-polynomial—the roots of which decide about stability—is subjected to iterative discretization by means of pre-warped bilinear transformation. Then, a linear and a quadratic interpolation are applied to obtain the associated characteristic polynomial with integer powers. The roots of the associated characteristic polynomial are closely related to the estimation of roots of the original characteristic quasi-polynomial which agrees with the system′s eigenvalues. Since the stability border is crossed by the leading one, the switching root locus is enhanced using the Regula Falsi interpolation method. Our methodology is implemented on—and verified by—a numerical bio-cybernetic example of the stabilization of a human-being′s movement on a controlled swaying bow. The advantage of the proposed novel algorithm lies in the possibility of the rapid computation of polynomial zeros by means of standard programs for technical computing; in the low level of mathematical knowledge required; and, in the sufficiently high precision of the roots loci estimation. The relationship to the direct search QuasiPolynomial (mapping) Rootfinder algorithm and computational complexity are discussed as well. This algorithm is also applicable for systems with non-commensurate delays.

## Introduction

The stability of Time Delay Systems (TDS) has been a challenging and intensively studied research topic over the past few decades [1–4]. It is, i.a., given by the fact that delay appears widely in many real-world systems—e.g. networked, mechanical, electrical, social, economical, etc. [5–7]. Moreover, delay can act as a factor that causes performance degradation and system instability; hence, the latencies and the after-effect phenomena should be considered in the models. Biological systems and human-body models are not unaffected; for instance, a robust estimator of neuronal interaction delays was proposed in [8]; the stabilization mechanism of unstable human quietstanding and motion was studied in [9–11]—where delays are, however, only marginally considered. The human nervous system with reflex delay can also be viewed as the cause of traffic jams [12]. Robust compensation of instability caused by delays for a human standing still was proposed by Asai, et al. [13]. Apart from the above-mentioned habitual studies that take fixed delay values into account; from the robustness point-of-view, it was desirable to also perform research on the influence of particular delay values on stability. This task gives rise to the notion of Delay Dependent Stability—(DDS), which means that the system remains stable in a finite set of delay intervals while taking into consideration fixed or variable system parameters.

Two basic families of DDS methods for computing the delay stability margins are paramount in the literature; namely, Time-domain Indirect and Frequency-domain Direct methods. The former group is based on the Lyapunov-Krasovskii or Lyapunov-Razumikhin approaches, and linear matrix inequalities [14–17]; the Jensen inequality approach [18]; semi-definite programming methods [19]; or the descriptor system approach [20,21]. Such methods suffer from significant computational burdens and provide purely theoretical—and rather conservative results [22]. However, they can deal with both constant and time-varying delays and are even applicable to non-linear systems in most cases. To highlight some important results regarding time-varying delay systems, the maximal allowed upper bounds of multiple time-varying delays expressed in terms of several linear matrix inequalities (LMIs) were investigated e.g. in [23,24]. In [25,26], these bounds were derived from feasible LMIs by employing MATLAB^®^ LMI Toolbox [27] to reach average consensus asymptotically; moreover, the rate of convergence was estimated. Compared to the presented paper, these methods provide only the upper bound, the given inequalities are obtained analytically and they are more mathematically involved; however, the domain of time-varying delays provides more general results. The latter family of frequency domain methods—usually better implementable and applicable—includes approaches based on various ideas, see e.g. [28] for their listing. Namely, from the historical point of view, methods based on the Rekasius substitution, giving rise to a delay-free Associated Characteristic Polynomial (ACP), constitute the group worthy of the highest research interest within this family [29–31]. An interesting solution can be obtained via the argument principle [32]. Besides this, Schur-Cohn methods [3,33]—or those eliminating exponential (delay) terms from the Characteristic QuasiPolynomial (CQP) due to its symmetry [28,34]—have also attracted great attention from the control scientific community. The crucial step of these procedures rests in the determination of all characteristic roots (system poles), i.e. the characteristic quasipolynomial zeros located exactly on the imaginary axis—which expresses the stability margin. However, only the rightmost subset of poles makes the system switch from/to stability/instability, respectively. Hence, let the corresponding delays be called switching delays. The necessary condition for the existence of roots crossing the imaginary axis is that the so-called root tendency is nonzero [30,34]. The frequency-domain direct methods, which are of a low—or even none conservatism and computationally acceptable in many cases, were successfully applied to investigate the stability of communications [28], power [35], electromechanical [36], logistic [37]; and many other systems with constant time delay. Note, however, that they cannot be directly applied to nonlinear TDS, or those with time-varying delays.

In spite of all the favorable features introduced above, a significant disadvantage of many frequency-domain direct methods is that they are only applicable to systems (or models) with commensurate delays where the higher order of commensuracy yields the higher complexity of the analytically derived relations [6]. Motivated by the above deficiency—and also by the endeavor to use easily-accessible standard programs for technical computing (e.g. MATLAB^®^) by engineers and practitioners, this paper focuses on deriving a computationally simple—yet sufficiently rapid and accurate gridding algorithm for searching for delay margins in both commensurate and non-commensurate TDS with an arbitrary number of independent constant delays. Although the method is not derived analytically in terms of exact analytic formulas, but rather, in a numerical way, the techniques used enable one to retain a very low conservatism level and to reach a high degree of precision; i.e., a discrete time reformulation of the CQP by means of the iterative use of the bilinear transformation with pre-warping [38], followed by the utilization of linear interpolation to obtain the integer-powered ACP, means that the zeros can easily be computed. In addition, a linear, Regula Falsi (RF), interpolation concept is then used for the eventual switching delay estimation.

In this contribution, the designed algorithm is applied to the stability delay margin analysis of a control system with a human on a swaying bow [39]. The skater balances on a hemispheric base and remotely steers the power input to the servo that causes the angle deviation from the bow horizontal position; and consequently, the angle between the skater and the bow symmetry axis is the controlled system output. For fixed controller parameters’ values, switching delays decide about the marginal balance values that cause stability/instability feedback behavior. The model is specified, i.a., by the fact that the delay-free feedback system is unstable—which indicates that the delay margins have upper and lower bounds. Moreover, the unstable controlled plan includes two independent delays that can generally be viewed as non-commensurate ones. Note that a problem of stabilizing the skater moving on a skateboard affected by reflex delay (with a different model of the dynamics) was recently solved in [40].

The efficiency and asymptotical complexity of the proposed algorithm is benchmarked against the possibility of seeking out switching delays by using the direct QuasiPolynomial mapping Rootfinder (QPmR) algorithm [41–43] that proved to be suitable for finding the quasipolynomial zeros within a selected complex plane rectangular region. A detailed example of the use of the algorithm, which provides the reader with real time requirements and the accuracy obtained in comparison to trial-and-error switching delays search procedures, is introduced for illustration as well.

Thorough the paper, ℂ, ℝ, ℕ, ${\mathbb{N}}_{\text{0}}$ denote the set of, respectively, complex numbers, real numbers, positive and non-negative integers. The *n*-dimensional Euclidean space is denoted as ${\mathbb{R}}^{n}$ and let ${\mathbb{R}}_{0+}^{n}:=\left\{a=\left(a_{1},a_{2},\ldots ,a_{n}\right)\in {\mathbb{R}}_{}^{n}|a_{i}\geq 0,i=1,2,\ldots ,n\right\}$. For s∈ℂ, Re(*s*) and Im(*s*) denote, respectively, the real part and imaginary part of *s*. The empty set is denoted as ∅. The notation *d*(*s*|*a*) means a polynomial in variable *s*, the coefficients of which are determined by the parameter *a* under some particular operation.

## Methods

### The general TDS model and its spectrum

Let,

G(s)=n(s,τ)/d(s,τ) (1)

stand for the Laplace transfer function in s∈ℂ of a single-input single-output linear time-invariant TDS with multiple delays as a frequency-domain model where *n*(*s*, **τ**), *d*(*s*, **τ**) are quasipolynomials of the general form: $x\left(s,\tau \right)=s^{n}+\sum_{i=0}^{n}\sum_{j=1}^{h_{i}}x_{ij}s^{i}\text{exp}\left(-s\sum_{k=1}^{L}\lambda_{ij,k}\tau_{k}\right)$, in which $\tau =\left(\tau_{1},\tau_{2},\ldots ,\tau_{L}\right)\in {\mathbb{R}}_{+}^{L},L>1$, represent independent delays: $\lambda_{ij,k}\in {\mathbb{N}}_{0}$ and $x_{ij}\in \mathbb{R}$. Let $\;x_{a}\left(s,\tau \right)=1+\sum_{j=1}^{h_{n}}x_{nj}\text{exp}\left(-s\sum_{k=1}^{L}\lambda_{nj,k}\tau_{k}\right)$ be the associated exponential polynomial related to *x*(*s*, **τ**). If, $\;d_{a}\left(s,\tau \right)\in \mathbb{R}$, then the system defined in Eq (1) is designated as retarded; otherwise, the system is of a neutral type.

In the following section, we assume that there are no common roots of the numerator and denominator in Eq (1). Under this assumption, the system poles (characteristic roots) *s*_*i*_ agree with the zeros of *d*(*s*, **τ**), i.e. *s*_*i*_ ≔ {*s*:*d***(***s*, **τ**) = 0}, and thus: CQP ≡ *d*(*s*, **τ**). Let us denote the spectrum of the system as Σ ≔{*s*_*i*_}, the set of all zeros of *d*_*a*_(*s*,) as Σ_*a*_ and introduce some basic spectral properties that are utilizable in this study.

*Property 1* [4,6,44]: The system defined in Eq (1) has the following properties:

1. If nonzero *d*_*ij*_, *λ*_*ij*,*k*_ exist (where *d*_*ij*_ stands for the real-valued coefficient in *d*(*s*,) for *s*^*i*^ with *j*th delay) for some positive *τ*_*k*_ and some *i*, *j*, *k*, then the number of poles is infinite.
2. For a retarded system, for any β∈ℝ with |*β*| < ∞, only a finite number of poles are located in the half-plane Re(*s*) > *β*; while infinitely many others are located in Re(*s*) < *β*.
3. For a neutral system, a vertical chain of poles (*s*_*i*_) exists at *γ* ≔ max ReΣ_*a*_ such that lim_*i*→∞_ Re(*s*_*i*_) = *γ*, lim_*i*→∞_ Im(*s*_*i*_) = ∞ for |*s*_*i*_| < |*s*_*i+*1_| $\left|s_{i}\right|<\left|s_{i+1}\right|$.
4. Let $\bar{\gamma }:=\underset{\Delta \tau \to 0}{\text{sup}}\gamma$, then the number of poles with $\text{Re }s_{i}>\bar{\gamma }$ is always finite, and they are isolated.
5. Isolated poles behave continuously and smoothly with respect to **τ** on ℂ.

1. If one defines the spectral abscissa as the function: *α*(**τ**)≔**τ** ↦ sup Re Σ, for which the following holds true (*Property 2*): Function *α*(**τ**) may be nonsmooth and hence, not differentiable; e.g. in points with more than one real pole or conjugated pairs with the same maximum real part [45].
2. It is non-Lipschitz, for instance, at points where the maximum real part has a multiplicity greater than one [46].

Rephrasing Property 2 –especially the second paragraph, abrupt changes may occur in the value of *α*(**τ**); however, such cases are rather rare—in at most, a finite number of delay vector points.

### TDS stability

*Proposition 1* [3,4]: The system as in Eq (1) with a fixed **τ** is exponentially stable if *α*(**τ**) < 0 for retarded systems; and if *α*(**τ**) < −*ε*, *ε* > 0 for neutral systems.

Note that, if *γ* < 0 (including infinity); then a TDS is so-called formally stable and Proposition 1 can collapse into the condition *α*(**τ**) < 0. The value of *γ* is, moreover, sensitive to even infinitesimal delay deviations (see item 4) of Property 1). This feature gives rise to the notion of strong stability—which means that a system is preserved from formal instability under small delay changes [4] (i.e. $\bar{\gamma }<0)$. The necessary and sufficient strong stability condition can be expressed as:

$$\sum_{j=1}^{h_{i}}\left|d_{nj}\right|<1$$ (2)

To avoid dealing with a potentially infinite chain of vertical system poles reaching or crossing the imaginary axis, retarded and/or strongly stable neutral systems satisfying Eq (2) are the only ones to be further considered in this study.

Let us now define some other useful notions regarding TDS stability with respect to delay values. A TDS is said to be DDS, if it is exponentially stable for some delay vectors values: $\tau_{i}\in {\mathbb{R}}_{0+}^{L}\backslash \infty$, constituting open sets (regions) of stabilizing delays. The root tendency (*RT*) is defined as:

RT=sgn Re{grad s(τ)} (3)

Crossing delays **τ**_*c*,*i*_, and the corresponding crossing frequencies, *ω*_*c*,*i*_, satisfy:

$${d\left(\text{j}\omega ,\tau \right)|}_{\begin{array}{l} \omega =\omega_{c,i}\\ \tau =\tau_{c,i} \end{array}}=0$$ (4)

For switching delays ${\bar{\tau }}_{i}$, and the corresponding switching frequencies ${\bar{\omega }}_{i}$, the condition in Eq (4) holds—and moreover, the corresponding pole (${\bar{s}}_{i}=\text{j}{\bar{\omega }}_{i}$) on the imaginary axis is the leading (furthest right) one; i.e. $\alpha \left({\bar{\tau }}_{i}\right)=\text{Re }{\bar{s}}_{i}=0$, and the imaginary axis is crossed with a non-zero velocity [17,30,47], i.e.:

$$RT{|}_{\begin{array}{c} \begin{array}{l} \tau ={\bar{\tau }}_{i}\\ \;s={\bar{s}}_{i} \end{array} \end{array}}\neq 0$$ (5)

Let us denote the set of all switching delays and frequencies as: $\bar{\text{T}}$ and $\bar{\Omega }$, respectively. It is evident from Proposition 1, that stability may only be violated at switching delays, while poles cross the imaginary axis at switching frequencies. This is the key idea of the direct frequency-domain methods cited above. Apart from the cases listed in Property 2, such an imaginary axis crossing is smooth.

### Model of a human being on a controlled swaying bow

**Fig 1:**
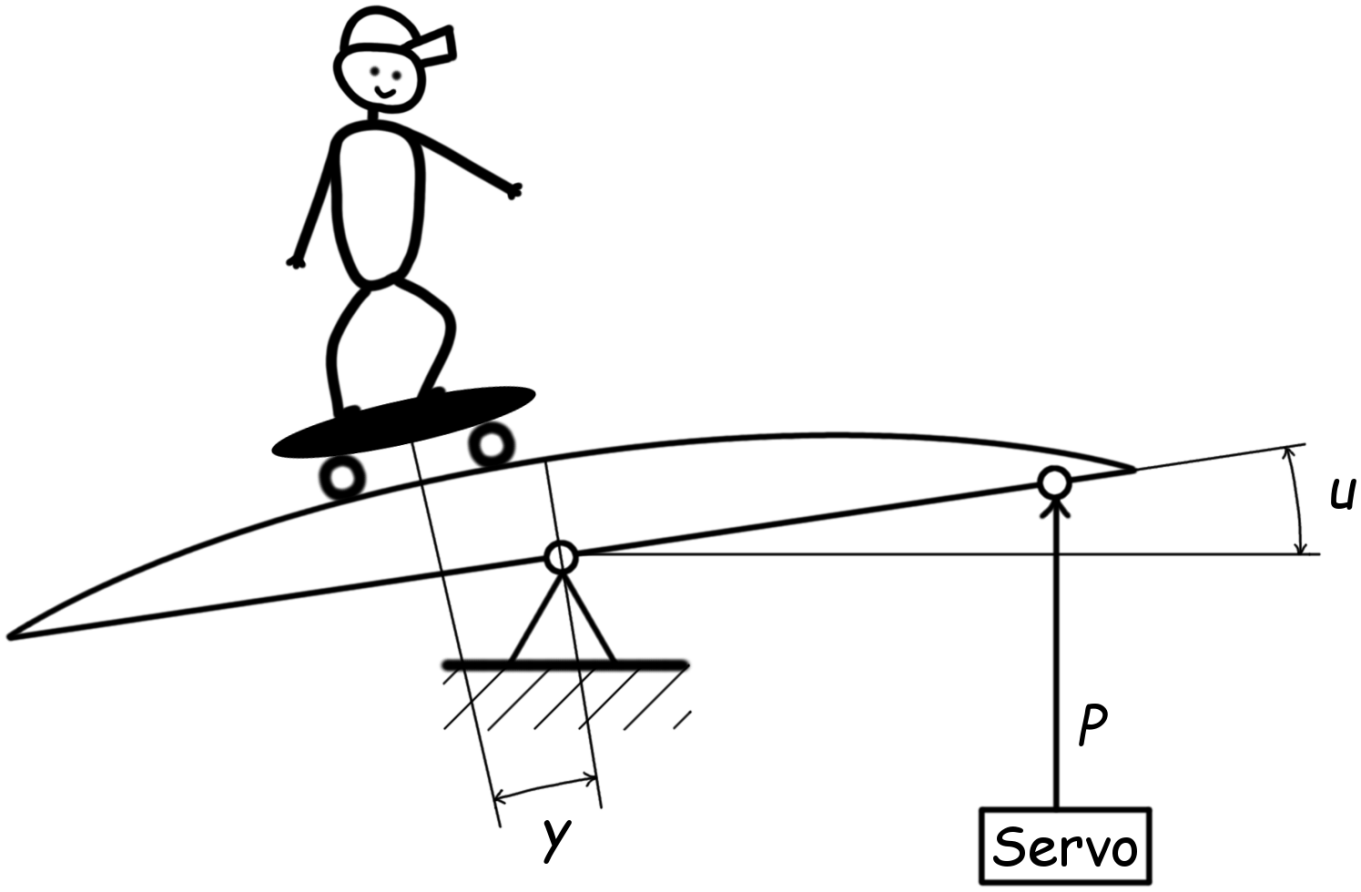
A skater on a remotely controlled swaying bow. The skater can send remote signals to the servomechanism (the rectangle labeled as Servo) that exerts power *P* in order to cause a horizontal-angle input deviation of *u*. The horizontal asymmetry yields the deviation of angle *y* from the bow symmetry axis—which is then taken as the system output.

In this study, we are concerned with a DDS stability analysis of a control system with a model of a (human) skater on a remotely-controlled swaying bow, see Fig 1. The skater controls the power input, *P*(*t*) to the servo—giving rise to the angle deviation, *u*(*t*) from the horizontal position, and consequently, the angle between the skater and the bow symmetry axis, *y*(*t*) emerges.

If friction is neglected, the following transfer function: (*u*(*t*) → *y*(*t*)) can be written:

$$G\left(s\right)=\frac{Y\left(s\right)}{U\left(s\right)}=\frac{b\text{exp}\left(-\left(\tau_{1}+\tau_{2}\right)s\right)}{s^{2}\left(s^{2}+a\text{exp}\left(-\tau_{2}s\right)\right)}$$ (6)

for some particular bow parameters, where delay *τ*_1_ expresses the skater’s reaction time, and *τ*_2_ means the latency. Nominal controlled system parameters and delay values may be read for instance as: *a* = −1, *b* = 0.2, *τ*_1_ = 0.3, *τ*_2_ = 0.1, as given in [39]. It can be computed that, for such parameters, the controlled TDS is unstable since it has the spectral abscissa value of *α* = 0.9534.

Let us now assume that the habitual simple negative control feedback loop is applied to a plant given in Eq (6), to restore stability. Let the control loop be equipped with a finite-dimensional linear controller governed by the transfer function:

$$C\left(s\right)=\frac{\sum_{i=0}^{3}q_{i}s^{i}}{s^{3}+\sum_{i=0}^{2}p_{i}s^{i}}$$ (7)

which gives the following retarded CQP:

$$d\left(s,\left(\tau_{1},\tau_{2}\right)\right)=s^{2}\left(s^{2}+a\text{ exp}\left(-\tau_{2}s\right)\right)\left(s^{3}+\sum_{i=0}^{2}p_{i}s^{i}\right)+b\text{ exp}\left(-\left(\tau_{1}+\tau_{2}\right)s\right)\left(\sum_{i=0}^{3}q_{i}s^{i}\right)$$ (8)

An optimal spectral abscissa minimizing procedure for the above introduced parameters, see [48], yields the result:

$$\begin{array}{l} p_{2}=469418.2,p_{1}=640264.2,p_{0}=10560107,q_{0}=5617613,q_{1}=26247749,\;q_{2}=106523133,\\ q_{3}=8222650 \end{array}$$

By considering this fixed setting, the nominal spectral abscissa of the control feedback loop is read as follows: *α*((0.3,0.1)) = −1.4454; while the delay-free case is: *α*((0,0)) = 0.1323. This means that system stability is delay dependent, and some switching delays might be found for fixed controlled plant and controller parameters.

### Discretization tools and techniques

This section intends to provide the reader with the basics of computational discretization techniques and implements useful for the course of the below proposed algorithm.

#### ACP polynomial approximation

The DDS algorithm introduced further on, in the results section, is designed to be practically implementable by means of most standard programs and utilities for scientific computing—without the necessity of the use of special software. Motivated by this, the first crucial step is to suggest a sufficiently accurate polynomial approximation of the CQP, resulting in the ACP, the leading zeros of which are close to those of the CQP, and can easily be computed.

The concept is based on the successive (iterative) discretization of the CQP, depending on the current leading pole estimation; i.e., a digital-filter-like implementation of the CQP is obtained. The Tustin (or bilinear) transformation is:

$$s\to \frac{2}{T}\frac{1-q}{1+q}$$ (9)

and represents a standard technique for the corresponding designing of the digital filter to a continuous-time model, where *T* is the sampling period; and *q* expresses the shifting operator that agrees with *z*^-1^ in the *z*-transform [9]. Let us recall that the mapping given in Eq (9) stems from the Taylor series expansion of the mutual relation between the *s*-plane and the *z*-plane:

$$z=\text{exp}\left(sT\right)\Leftrightarrow s=\frac{1}{T}\text{ln }z$$ (10)

The crucial feature of this mapping is that it preserves stability, i.e. the poles in the complex left-half *s*-plane fall into the interior of the unit circle in the *z*-plane, and vice versa.

However, it is also known that—for finite-dimensional systems—the transformation given by Eq (9) does not preserve frequencies (i.e. so-called frequency warping occurs). The mutual relations are:

$$\omega =\frac{2}{T}\text{tan}\left(\frac{\omega_{d}T}{2}\right)\Leftrightarrow \omega_{d}=\frac{2}{T}\text{arctan}\left(\frac{\omega T}{2}\right)$$ (11)

where *ω*, *ω*_*d*_ stand for the frequency of the continuous-time model and the corresponding digital filter, respectively. Our intention is to make the ACP a digital implementation of the CQP, for which the imaginary part of the leading (dominant) root—after the use of the substitution as per Eq (10)—matches that of the CQP; which, i.a., means that both the corresponding switching imaginary poles might coincide. Hence, the CQP ought to be rescaled so that *ω*_*d*_—calculated from Eq (11), equals the desired (original, non-scaled) *ω*. This idea yields the eventual pre-warping filter design mapping as follows:

$$s\to \frac{\omega }{\text{tan}\left(\omega T/2\right)}\frac{1-q}{1+q}$$ (12)

The task of the choice of *T* is not unambiguous. With respect to delta models and derivative discretization, the value of *T* in Eqs (9) or (12) should be sufficiently small; while, on the contrary—the lower *T* is, the higher ACP degree is obtained. Note that for the *z*-transform, a recommendation for periodic systems has been given as: T = [0.2/*ω*_0_,0.5/*ω*_0_], where *ω*_0_ expresses the frequency of undumped oscillations [49]. Adopting the basic knowledge about a second order finite-dimensional model: *ω*_0_ = |*s*_0_|, where *s*_0_ means a pole from the conjugate pair, one can write the interval as:

$$T=\left[0.2{\left|s_{0}\right|}^{-1},0.5{\left|s_{0}\right|}^{-1}\right]$$ (13)

Note that for infinite-dimensional systems; *s*_0_ can be taken as the dominant pole.

The specific feature of TDSs is that the CQP includes exponential terms that are not subjected to either Eqs (9) or (12). In fact, the bilinear transformation acts only on *s*-powers in the CQP corresponding to output derivatives in the system model. Exponentials are processed by natural shifting:

$$\text{exp}\left(-\vartheta s\right)X\left(s\right)\hat{=}x\left(t-\vartheta \right)\hat{=}q^{\vartheta /T}x\left(k\right)\hat{=}z^{-\vartheta /T}X\left(z\right)$$ (14)

#### Switching delay estimation

Once the ACP is found for each grid node given by the delay discretization, i.e. for a particular value of **τ**, its leading zero is calculated—thus expressing the estimation of the leading zero of the CQP. As the gridding may be very sparse, the imaginary axis can usually be leaped over. A smoother switching delay estimation—and thus, that of the switching pole, can be made by the linear RF interpolation as follows:

$${\bar{\tau }}_{k}\left(\tau_{k,j},{\hat{s}}_{j},{\hat{s}}_{j+1}\right)=\tau_{k,j}-\text{Re }{\hat{s}}_{j}\frac{\tau_{k,j+1}-\tau_{k,j}}{\text{Re }{\hat{s}}_{j+1}-\text{Re }{\hat{s}}_{j}}$$ (15)

where ${\bar{\tau }}_{k}$ is the estimation of the *k*th element of the switching delay vector; *τ*_*k*,*j*_, *τ*_*k*,*j+*1_ stand for the delay elements in the *j*th and (*j* + 1)th nodes, respectively; and ${\hat{s}}_{j},{\hat{s}}_{j+1}$ represents the zeros of the ACP in the corresponding nodes. To rephrase, Eq (15) approximates the zero of the function: $\text{Re }\hat{s}\left(\tau_{k}\right)$. Let us recall that the necessary stability switching condition formulated in Eq (5) must hold for the eventual value of $\bar{\tau }$.

### QPmR overview

The QPmR mapping algorithm was designed to compute all quasipolynomial zeros located in a given region in ℂ. Its leading idea is as follows: Let, *s* = *β* + j*ω*, β,ω∈ℝ, and thus split the CQP into two real functions *R*(*β*,*ω*)≔Re{*d*(*β* + j*ω*,)}, *I*(*β*,*ω*)≔Im{*d*(*β* + j*ω*,)}. The region of interest is covered by a rectangular mesh grid: $\left(\beta_{0},\beta_{1},\ldots \beta_{k_{\text{max}}}\right)\times \text{j}\left(\omega_{0},\omega_{1},\ldots \omega_{l_{\text{max}}}\right)$. Then, the zero of the CQP can be found by the intersection of the curves given by the solutions of: *R*(*β*,*ω*) = 0, *I*(*β*,*ω*) = 0, within the gridded region. The accuracy of the zeros is subsequently increased by the Newton method.

The original version (v.1) was designed by Vyhlídal and Zítek [41], and implemented in Matlab based on the use of the Symbolic Math Toolbox. However, the toolbox led to a relatively large computational time. The QPmR was improved in the consecutive work [42], where optimization via the reduction of the scanned regions was implemented. This was achieved by using the argument principle and spectrum distribution analysis [50]. As a result, a significant reduction of computational time was obtained; however, the complexity of the algorithm increased rapidly. The enhanced algorithm was implemented once again in Matlab, as an advanced QPmR (aQPmR). The up-to-date version (v.2) of the algorithm was published in [43]. The authors avoided the use of the Symbolic Math Toolbox—and included the recursive grid density adaptation, which resulted in at least twice as fast computing rates.

Naturally, the QPmR can be utilized for switching delay searches, either as a trial-and-error procedure when directly searching within the given delay range, or as a tool for the evaluation of CQP zeros, instead of the polynomial approximation introduced above. Note however, that the attention of this paper is, i.a., devoted to achieving the goal of avoiding the necessity of using special software tools. Our intention is to employ the QPmR as a benchmark for the algorithm presented herein.

## Results and discussion

Prior to the presentation of the proposed DDS algorithm and its implementation on stability margin detection when remote controlling a skater on a swaying bow, some additional methods have to be derived and computational ideas formulated.

### Integer-powers polynomial interpolation

The discrete-time formulation of exponentials given by Eq (14) generally results in: ϑ/T∉${\mathbb{N}}_{0}$; thus, it is necessary to apply an interpolation:

$$z^{-\vartheta /T}\approx a_{0}z^{-\left\lfloor \vartheta /T\right\rfloor }+a_{1}z^{-\left(\left\lfloor \vartheta /T\right\rfloor +1\right)}+a_{2}z^{-\left(\left\lfloor \vartheta /T\right\rfloor +2\right)}+\ldots$$

in order to acquire a polynomial with integer powers. A possible way to cope with this task is presented in the following theorem.

*Theorem 1*: A term $z^{-\left(n+m\right)}\in \mathbb{C}$, where $n\in {\mathbb{N}}_{0}$, 0 < *m <* 1 can be approximated in the neighborhood of arbitrary $z_{0}^{}\in \mathbb{C}$ as:

1. Linear interpolation:
  $$z^{-\left(n+m\right)}\approx \left(1-m\right)z_{0}^{-m}z^{-n}+mz_{0}^{-m+1}z^{-\left(n+1\right)}$$ (16)
2. Quadratic interpolation:
  $$z^{-\left(n+m\right)}\approx 0.5\left(2-m\right)\left(1-m\right)z_{0}^{-m}z^{-n}+m\left(2-m\right)z_{0}^{-m+1}z^{-\left(n+1\right)}+0.5m\left(m-1\right)z_{0}^{-m+2}z^{-\left(n+2\right)}$$ (17)
  The proof of Theorem 1 is provided in the Appendix.

*Corollary 1*: If Eq (16) is performed at *z*_0_ = 1, which agrees with *s*_0_ = 0 in the *s*-plane, the natural and intuitive result: *z*^−(*n*+*m*)^ ≈ (1 − *m*)*z*^−*n*^ + *mz*^−(*n*+1)^ is obtained—which expresses the linear distribution in the accordance with the “closeness” of *m* to 0 or 1.

In our algorithm, the value *z*_0_ is selected as the leading (dominant, right-hand most) pole *s*_0_ subjected to Eq (10).

### Successive leading pole estimation

Once the ACP in *z*^-1^ is found by using Eqs (9) or (12), (14), (16) or (17), its dominant root can be computed simply by means of standard engineering computing software, which is a benefit of the algorithm presented below. In order to obtain a more precise estimation, the iterative procedure rather than a single-step calculation is used. It initially adopts the result obtained from the preceding delay discretization grid node (or more precisely, from the nearest previously solved node)—providing the preliminary estimation of the particular dominant pole: ${\hat{s}}_{0,\left(1\right)}$. The choice of *ω* in Eq (12) and the time period in Eq 13 in its *i*th iteration is given by: ${\hat{s}}_{0,\left(i\right)}$ and the interpolation introduced in Eqs (16) or (17) is performed at ${\hat{z}}_{0,\left(i\right)}$—resulting from ${\hat{s}}_{0,\left(i\right)}$ by means of Eq (10), which gives rise to the ACP as $\hat{d}\left(z^{-1}|\tau ,{\hat{s}}_{0,\left(i\right)}\right)=\sum_{i=0}^{\hat{n}}{\hat{d}}_{i}\left(\tau ,{\hat{s}}_{0,\left(i\right)}\right)z^{-i}$ where $\tau ,{\hat{s}}_{0,\left(i\right)}$ stand for the parameters′ values that determine the polynomial coefficients. In every single iteration step, the updated leading root ${\hat{z}}_{0,\left(i+1\right)}$ of the ACP is subjected to Eq (10) so as to consequently update: ${\hat{s}}_{0,\left(i+1\right)}$. In fact, the root closest to the preceding one is evaluated as the leading one for the next iteration step. The iterative procedure for the particular node of the grid is terminated if $\left|{\hat{s}}_{0,\left(i+1\right)}-{\hat{s}}_{0,\left(i\right)}\right|<\varepsilon$ for a selected *ε* > 0.

The motivation for the iterative calculation of the ACP in the neighborhood of the currently found leading pole can be explained as follows: The pole furthest right of the CQP spectrum is the only decisive one with respect to TDS stability—as clear from Proposition 1. Let this pole: *s*_0_, be isolated and known for a particular **τ**_0_ exactly. Due to Property 1, for any *ε* > 0, there exists *δ* > 0 such that if: **τ**_1_ = **τ**_0_ + Δ**τ**,‖Δ**τ**‖ < *δ*, the corresponding root *s*_1_≔{*s* → min|*s* −*s*_0_|:*d*(*s*, **τ**_1_) = 0} satisfies |*s*_*1*_ −*s*_0_| < *ε*. Hence, for a sufficiently small; roots *s*_0_ and *s*_1_ are close to each other. Let us assume the ACP as: $\hat{d}\left(z^{-1}|\tau_{1},s_{0}\right)$ and calculate its leading zero ${\hat{z}}_{1,\left(1\right)}$ (i.e. that with the maximum modulus). It is supposed that ${\hat{s}}_{1,\left(1\right)}$ resulting from ${\hat{z}}_{1,\left(1\right)}$ via the exact mapping given in Eq (10) is located in the vicinity of *s*_0_ with $\left|s_{1}-s_{0}\right|>\left|s_{1}-{\hat{s}}_{1,\left(1\right)}\right|$. Now, by the iterative calculation of ${\hat{d}}_{\left(i\right)}\left(z^{-1}|\tau_{1},s_{1,\left(i\right)}\right)$, the corresponding *s*-plane maps of their zeros, ${\hat{s}}_{1,\left(i+1\right)}$, should satisfy $\left|s_{1}-{\hat{s}}_{1,\left(i\right)}\right|>\left|s_{1}-{\hat{s}}_{1,\left(i+1\right)}\right|$ and hence, there exists a descending sequence: ${\left\{\left|d_{\left(i\right)}\left({\hat{s}}_{1,\left(i\right)},\tau_{1}\right)\right|\right\}}_{i=1}^{i_{\text{max}}}$ which converges for a suitable choice of *T*. A problem can emerge from Property 2 if $\left|{\hat{s}}_{1,\left(i+1\right)}-{\hat{s}}_{1,\left(i\right)}\right|>\delta$ for some *δ* > 0 and any Δ**τ** due to the spectral abscissa discontinuity; however, such cases are rare and can be omitted in practice—since, for instance, the imaginary axis cannot be crossed by a multiple real pole [30,50]. Moreover, only strongly stable systems are taken into consideration.

### Switching delay search algorithm

The proposed gridding switching delay search algorithm poses the main goal of this study and, it is summarized below by using ordered steps and a flowchart. The framework of the algorithm consists of the following steps: initialization, ACP computation and seeking its zeros, as well as switching delays and poles′ values enhancement.

Firstly, the $L\times N\in {\mathbb{N}}^{2}$ mesh grid of delays within the selected ranges must be created, hence: *τ*_*k*,*j*+1_ = *τ*_*k*,*j*_ + Δ*τ*_*k*,*j*_, *τ*_*k*,0_ = 0, *k* = 1…*L*, *j* = 0…*N* − 1. The discretization step Δ*τ*_*k*,*j*_ has to be chosen according to the minimum acceptable estimation error. Note, however, that numerical results have shown that this value may be approximately 10^3^-times higher than eventual obtained accuracy. The counter of found switching delays (poles) are to be initialized as well, *i* = 1, and select the admissible convergence error while computing the ACP, *ε* > 0. The initial leading pole, ${\hat{s}}_{0}=s_{0}$, can easily be calculated exactly for **τ** = **0** as the rightmost zero of the polynomial *d*(*s*,**0**).

In each node of the grid (implemented as nested loops over *τ*_*k*_), the iterative polynomial estimation of the CQP, i.e. the ACP, is computed. Once the final estimations of the dominant poles ${\hat{s}}_{0}$, ${\hat{s}}_{1}$ in two subsequent grid nodes are found, see the preceding section for details, the test whether the imaginary axis has been crossed is executed. If it passes, the RF as in Eq (15) is successively performed to receive ${\bar{\tau }}_{k}$ and updated values of ${\hat{s}}_{0}$, ${\hat{s}}_{1}$ are computed by iterations for each *k*. The eventual delay vector $\hat{\bar{\tau }}$ has to be subjected to the *RT* condition test formulated in Eq (5). Estimations of switching delays ($\hat{\bar{\text{T}}}$), switching poles ($\hat{\bar{\Sigma }}$) and their imaginary parts ($\hat{\bar{\Omega }}$), i.e. switching frequencies, are obtained as the algorithm outputs. The algorithm summary is as follows (*Algorithm 1*):

1. For a neutral TDS—verify the strong stability condition in Eq (2). If it does not pass, terminate the algorithm.
2. For the given *d*(*s*, **τ**), define the mesh grid: *τ*_*k*,*j*+1_ = *τ*_*k*,*j*_ + Δ*τ*_*k*,*j*_, *τ*_*k*,0_ = 0, *k* = 1…*L*, *j* = 0…*N* − 1 for a selected delay range, initialize the counter: *i* = 1 and choose *ε* > 0. Set: $\hat{\bar{\text{T}}}=\hat{\bar{\Sigma }}=\hat{\bar{\Omega }}=\emptyset$.
3. Compute:
  $${\hat{s}}_{0,\ldots ,0}=s_{0,\ldots ,0}:=\left\{\text{arg max Re }s:d\left(s,0\right)=0\right\}$$ (18)
4. For: (*j*_1_ = 0…*N* − 1, for (*j*_2_ = 0…*N* − 1… (for *j*_*L*_ = 0…*N* − 1 do steps 5–11)).
5. If: *j*_1_ = *j*_2_ = … = *j*_L_ = 0, the inner loop is finished; if not, define:
  $$M:=\text{max}\left\{k:j_{k}\neq 0\right\}$$ (19)
  and set:
  $$\begin{array}{l} \tau =\left(\tau_{1,j_{1}},\tau_{2,j_{2}}\ldots ,\tau_{L,j_{L}}\right)\\ {\hat{s}}_{old}={\hat{s}}_{0}={\hat{s}}_{j_{1},\ldots ,j_{M-1},j_{M}-1,0\ldots 0} \end{array}$$ (20)
6. Compute: $\text{ACP}\equiv \hat{d}\left(z^{-1}|\tau ,{\hat{s}}_{0}\right)$ according to Eqs (9) or (12), (14), (16) or (17). Find its roots (*z*_*i*_) and subject them to Eq (10), which yield *s*_*i*_. Define:
  $${\hat{s}}_{1}:=\text{arg min}\left|s_{i}-{\hat{s}}_{0}\right|$$ (21)
7. While $\left|{\hat{s}}_{1}-{\hat{s}}_{0}\right|\geq \varepsilon$, set ${\hat{s}}_{0}:={\hat{s}}_{1}$; and then go to step 6.
8. Set: ${\hat{s}}_{new}={\hat{s}}_{j_{1},\ldots ,j_{L}}={\hat{s}}_{j_{1},\ldots ,j_{M-1},j_{M},0\ldots 0}={\hat{s}}_{1}$; if $\text{sgn}\left(\text{Re }{\hat{s}}_{new}\right)=\text{sgn}\left(\text{Re }{\hat{s}}_{old}\right)$, the inner loop is finished (see step 4); otherwise, *i* = *i* + 1 and go to step 9 (RF).
9. Set ${\bar{\tau }}_{M}={\bar{\tau }}_{M}\left(\tau_{M,j_{M}-1},{\hat{s}}_{old},{\hat{s}}_{new}\right)$ where function ${\bar{\tau }}_{M}$ is defined in Eq (15) and perform the cycle given in step 10.
10. For: *k = M* − 1,…,1 do: If *j*_*k*_ = 0, set ${\bar{\tau }}_{k}=\tau_{k,0}$ and go back to step 10; or else, set ${\hat{s}}_{0}={\hat{s}}_{new}$ and
  $$\tau_{old}=\left(\tau_{1,j_{1}},\ldots ,\tau_{k,j_{k}-1},{\bar{\tau }}_{k+1},\ldots ,{\bar{\tau }}_{M},0,\ldots ,0\right),\tau =\left(\tau_{1,j_{1}},\ldots ,\tau_{k,j_{k}},{\bar{\tau }}_{k+1},\ldots ,{\bar{\tau }}_{M},0,\ldots ,0\right)$$ (22)
  and then compute the leading root ${\hat{s}}_{1}$ from $\hat{d}\left(z^{-1}|\tau_{old},{\hat{s}}_{0}\right)$ as in steps 6–7. Update values ${\hat{s}}_{old}={\hat{s}}_{0}={\hat{s}}_{1}$ and find the leading root ${\hat{s}}_{1}$ from $\hat{d}\left(z^{-1}|\tau ,{\hat{s}}_{0}\right)$ according to the cycle in steps 6 and 7 again, and update the value ${\hat{s}}_{new}={\hat{s}}_{1}$. Then: ${\bar{\tau }}_{k}={\bar{\tau }}_{k}\left(\tau_{k,j_{k}-1},{\hat{s}}_{old},{\hat{s}}_{new}\right)$. When the loop is finished, consolidate ${\bar{\tau }}_{i}=\left({\bar{\tau }}_{1},\ldots {\bar{\tau }}_{M},0,\ldots 0\right)$ and go to step 11.
11. Update ${\hat{s}}_{0}={\hat{s}}_{new}$, $\hat{\bar{Τ}}=\hat{\bar{Τ}}\cup {\bar{\tau }}_{i}$. Compute the leading zero iteratively: ${\bar{s}}_{1}={\hat{s}}_{1}$ from $\hat{d}\left(z^{-1}|{\bar{\tau }}_{i},{\hat{s}}_{0}\right)$ in accordance with steps 6–7. Set $\hat{\bar{\Sigma }}=\hat{\bar{\Sigma }}\cup {\bar{s}}_{1},\hat{\bar{\Omega }}=\hat{\bar{\Omega }}\cup \left|\text{Im }{\bar{s}}_{1}\right|$.
12. Outputs: $\hat{\bar{Τ}},\hat{\bar{\Sigma }},\hat{\bar{\Omega }}$.

**Fig 2:**
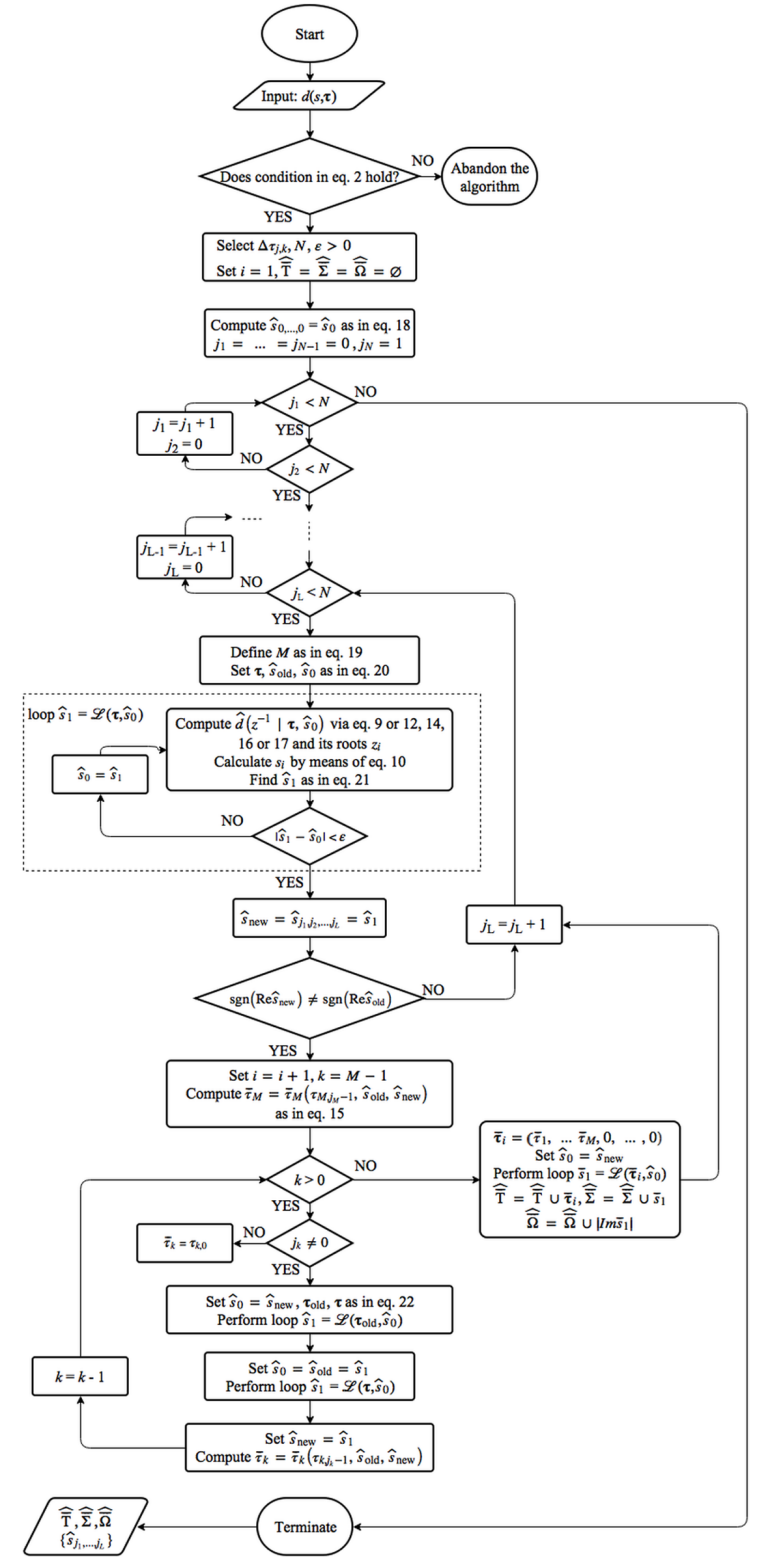
A Flowchart diagram of Algorithm 1. The flowchart diagram displays the designed DDS algorithm. Its step-by-step formulation is presented in Algorithm 1. The ellipse expresses the starting point, and the oval means the termination point. The rhomb, the trapezium and the square stand for the condition, the output and the execution block, respectively.

A flowchart of Algorithm 1, beneficial particularly for practitioners, is depicted in Fig 2.

### Application of the algorithm to the model of a skater

The particular example presented herein, is aimed at Algorithm 1′s accuracy and speed verification, and demonstrates its usability and applicability in bio-cybernetics, by means of the estimation of the stability switching delays for the control loop of a model of a skater on a controlled swaying bow (see Eq (6) and Fig 1) and equipped with the controller given by Eq (7). A set of tests includes the determination of a suitable value of *T*, the comparison of the use of the linear and quadratic interpolations is according to Eqs (16) and (17) respectively, and the demonstration of the pre-warping asset.

Prior to the presentation of the course of Algorithm 1 itself, let us perform some numerical tests to determine a suitable value of *T*.

#### Sampling period determination

Let a particular delay vector be chosen and fixed as: **τ** = (0.07,0.07), and let us parameterize *T* as:

$$T=\frac{1}{k_{T}\left|s_{0}\right|},k_{T}\geq 2$$ (23)

with respect to the upper bound of the condition given by Eq (13). Let us assume moreover, that the leading zero of *d*(*s*,(0.07,0.06)) is known exactly—the use of the QPmR with the precision of 10^−9^ gives the value: *s*_0_ = 0.024805739 ± 3.905723903i—which is used as the initial estimation. An accuracy comparison of leading pole estimations by means of the ACP described in steps 6 and 7 of Algorithm 1 is shown in Table 1. Both the first and second order interpolations as in Eqs (16) and (17) respectively are included—and, moreover, the beneficial impact of pre-warping governed by the Eq (11) is demonstrated. The error expresses the distance of the approximated leading pole from *s*_0_ = 0.007985448 ± 3.868912827i; found by the QPmR with the above-mentioned precision. The admissible iteration error was set to: *ε* = 10^−6^ (see step 1 of Algorithm 1) and the maximum acceptable number of iteration steps was set to 200. The eventual approximated leading pole loci are given to the reader in S1 Table.

**Table 1:** Leading pole estimation accuracy benchmark.

Method	*k*_*T*_	Error	*iter*_max_
Eqs (9) and (16)	2	7.025e-2	5
	3	3.109e-2	4
	5	1.112e-2	4
	10	2.79e-3	3
	20	6.99e-4	5
	25	4.52e-4	29
	100	-	Inf
Eqs (12) and (16)	2	2.86e-4	6
	3	1.28e-4	4
	5	4.6e-5	4
	10	1.2e-5	3
	20	3e-6	3
	25	1.0e-5	2
	30	3.6e-5	18
	100	-	Inf
Eqs (12) and (17)	2	2.86e-4	5
	3	1.28e-4	4
	5	4.6e-5	4
	10	1.2e-5	3
	20	3e-6	6
	25	4e-6	5
	30	1.9e-5	37
	100	-	Inf

There are the results of a comparison of the use of Eqs (9), (12), (16) and (17) in Algorithm 1, and applied to the control of a skater on a swaying bow for a fixed delay value and several values of *T*–see Eq (13). Error means the distance of computed leading poles from the exact analytic one found by the QPmR. The value of *iter*_max_ stands for the number of iterations within the successive polynomial leading root computation until the precision of *ε* is reached.

As can be seen from Table 1, the degree of precision is one to three orders better with the use of pre-warping than the bilinear transformation without it. Notice moreover, that it is necessary to perform a lower number of iterations to reach the desired *ε* for higher *k*_*T*_—yet, the value is bounded.

Generally, high values of *k*_*T*_ require enormous number of iterations—or even cause divergence—and, the error thus increases slightly. The growth of the error value can be explained as follows. Algorithm 1 uses the bilinear (Tustin) transformation as in Eqs (9) or (12) to get the associated polynomial, and the (back) transformation from the *s*-plane to the *z*-plane via the exponential formula as in Eq (10). Geometrically, the conformal mapping (Eq (9)) maps the closed left-hand side of the complex *s*-plane inside the circle in the *z*-plane with the radius of 1/*T*, i.e., linearly with respect to *k*_*T*_. The transformation according to Eq (10) provides the map inside the unit circle, i.e. the radius does not depend on the value of *k*_*T*_. From the operator point of view, the Tustin formula represents the trapezoidal (feedback-feedforward) form of the derivative approximation. It is closely related to the delta transform. Hence, intuitively, the shorter the step *T* is (i.e. the higher *k*_*T*_ is), the approximation more precise is. However, the latter formula agrees with the transition from the *z*-transform to the s-transform (or vice-versa). It is well know that the *z*-transform fails for a very small values of the time period *T* (for instance, transfer function poles from the transfer function in *s* collapse to the point 1 in the *z*-plane). Moreover, the division be a very small number induces numerical problems when computing. The algorithm has to respect a compromise between both the principles. Due the properties of the *z*-transform and because of the numerical representation of real numbers inside the computer, the value of *T* must be sufficiently high, i.e. the value of *k*_*T*_ can not be excessively high.

Only a slight improvement is achieved by means of a quadratic interpolation—which is, however, sacrificed by the necessity of more iteration steps. In fact, two leading roots of the ACP do not constitute a complex conjugate pair due to complex polynomial coefficients. It should be noted that the quadratic interpolation yields better mutual convergence of these roots with ascending *k*_*T*_.

The advantage of the iterative calculation of the ACP can be demonstrated for instance, by the evolution of the error $\left|{\hat{s}}_{1}-s_{0}\right|$—see steps 6 and 7 of Algorithm 1 for ${\hat{s}}_{1}$; and *s*_0_ stands for the exact dominant system pole—with *k*_*T*_ = 15 and pre-warping as: {23.7,5.1,5}·10^−6^. Note that *ω* in Eq (12) has been set as: $\text{Im }{\hat{s}}_{1}$.

It seems from Table 1 that the suitable value of *T* lies approximately in the interval *k*_*T*_ ∈ [10,25]. Now, let us perform the second test as follows: Let the leading zero of *d*(*s*,(0.06,0)) be known exactly and perform Algorithm 1 in a degenerate form for *τ*_1_ ∈ [0.06,0.07], *τ*_2_ ∈ [0,0.2] with Δ*τ* = 0.01. The numerical results are summarized in Table 2, where $\bar{\tau }$ expresses the switching delay estimation, *s*_0_ is the corresponding leading system pole calculated by the QPmR, the error means the minimum distance of $\bar{\tau }$ from the one found by the QPmR with the delay resolution of Δ*τ* = 10^−7^; and the number of iterations (*iter*_max_) expresses the mean value rounded to the nearest integer.

**Table 2:** Switching delay estimation benchmark.

Method	*k*_*T*_	$\bar{\tau }$	*s*_0_	Error	*iter*_max_
Eqs (9) and (16)	2	(7.00526, 7.77128)e-2	-5.8741e-3 + 3.840208i	3.269e-3	4
	3	(7.00726, 7.58688)e-2	-2.5627e-3 + 3.846954i	1.427e-3	5
	5	(7.00751, 7.49427)e-2	-9.170e-4 + 3.846954i	5.09e-4	3
	10	(7.00746, 7.45718)e-2	-2.324e-4 + 3.851742i	1.27e-4	3
	20	(7.00744, 7.44771)e-2	-6.99e-5 + 3.852092i	3.3e-5	11
	25	(7.00763, 7.44654)e-2	-4.52e-5 + 3.852128i	2.2e-5	14
	100	-	-	-	Inf
Eqs (12) and (16)	2	(7.00754, 7.44444)e-2	-5.7e-6 + 3.8522095i	2.7e-6	5
	3	(7.00747, 7.44445)e-2	-5.6e-6 + 3.8522096i	2.2e-6	4
	5	(7.00744, 7.44454)e-2	-5.6e-6 + 3.8522096i	2.1e-6	4
	10	(7.00743, 7.44456)e-2	-5.6e-6 + 3.8522096i	2.1e-6	3
	20	(7.00743, 7.44454)e-2	-5.4e-5 + 3.8522100i	2.0e-6	5
	25	(7.00744, 7.44455)e-2	-5.7e-5 + 3.8522093i	2.2e-6	34
	30	-	-	-	Inf
Eqs (12) and (17)	2	(7.00754, 7.44444)e-2	-5.7e-6 + 3.8522095i	2.7e-6	5
	3	(7.00747, 7.44445)e-2	-5.6e-6 + 3.8522096i	2.2e-6	4
	5	(7.00744, 7.44454)e-2	-5.6e-6 + 3.8522096i	2.1e-6	4
	10	(7.00743, 7.44456)e-2	-5.6e-6 + 3.8522096i	2.1e-6	3
	20	(7.00744, 7.44454)e-2	-5.8e-5 + 3.8522090i	2.2e-6	4
	25	(7.00732, 7.44455)e-2	-3.4e-5 + 3.8522136i	1.4e-6	18
	30	-	-	-	Inf

Algorithm 1 is tested when the initial CHP root is known exactly for *τ* ∈ (0.06,0). Delay values go through intervals *τ*_1_ ∈ [0.06,0.07], *τ*_2_ ∈ [0,0.2] with *Δ*τ = 0.01. The value of $\bar{\tau }$ stands for the eventual estimated switching delay vector; and *s*_0_ is the corresponding leading ACP root. Error means the minimum distance of $\bar{\tau }$ from the exact one. The number of iterations is rounded.

Again, it can be deduced from Table 2 that the use of pre-warping brings about a significant improvement of the switching delay estimation. The quadratic interpolation however, does not come up to expectations; nevertheless, it can reduce the number of iterations for the ACP in a specific range of *k*_*T*_ and results in a better mutual approach to the leading complex pair. In order to also include the time consumption factor—given mainly by the number of iterations, the eventual range of *k*_*T*_ for this example is approximately: *k*_*T*_ ∈ [3,15] for Eq (9) and *k*_*T*_ ∈ [3,20] for Eq (13)–which goes beyond the suggestion formulated in Eq (13) and stands somewhere between the z-transform and derivative discretization. Hence, after some additional numerical experiments for the switching delay search herein below, we finally decided to set a uniform *k*_*T*_ = 8. Let us recall that *T* is adapted according to Eq (23) in every single iteration step of the ACP calculation with respect to the leading pole estimation (${\hat{s}}_{0}$).

#### Switching delay computation

**Fig 3:**
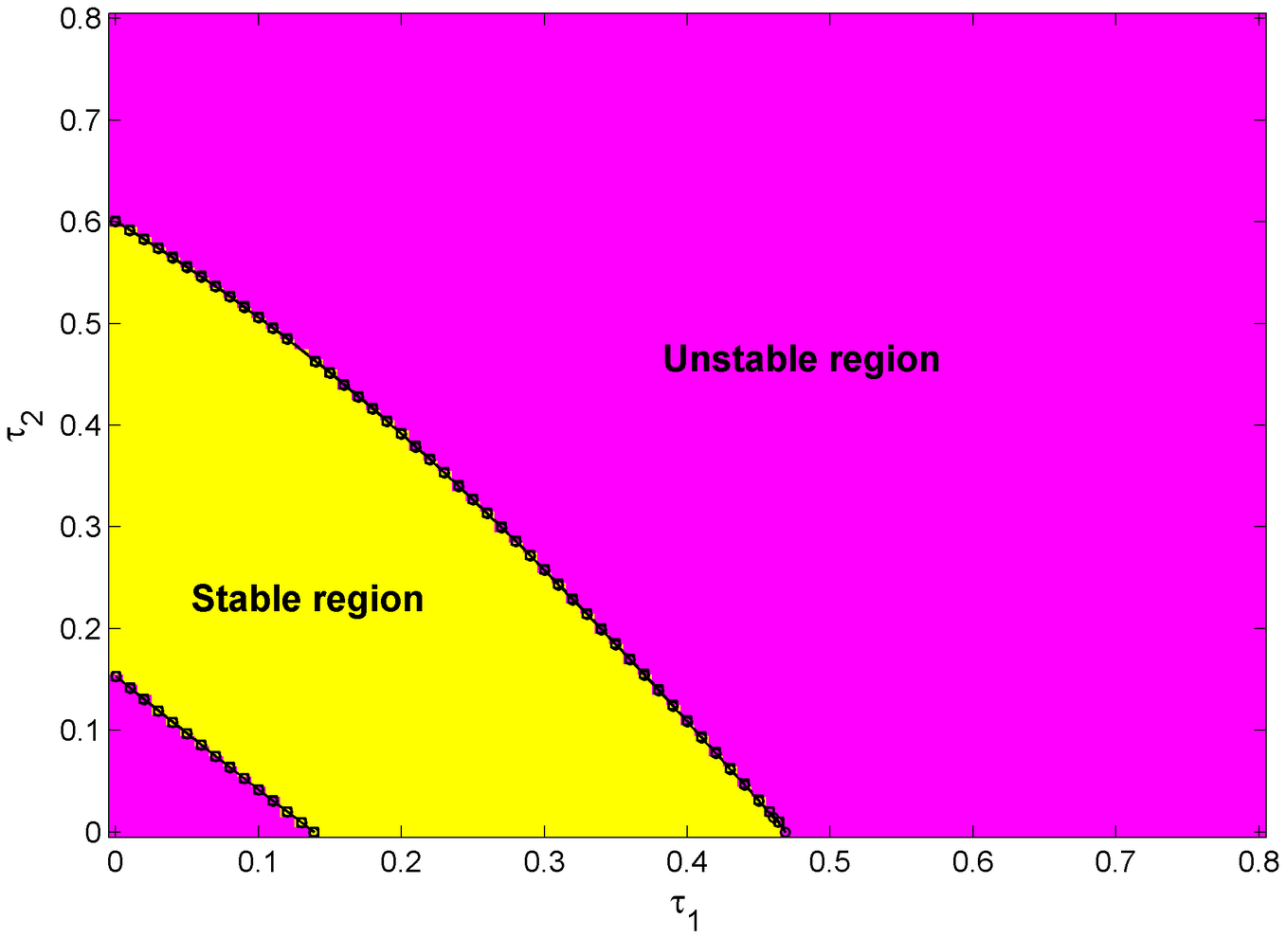
Switching delays found by Algorithm 1. Delay values computed by using pre-warping are indicated by circles and a joint via the solid line; the ones without pre-warping are represented by diamonds and joint by the dashed line. Results are back-grounded stability/instability regions computed by the QPmR with Δ*τ*_⋅_ = 0.01.

As the final numerical experiment—the complete course of Algorithm 1 is performed and its results are presented here below. Let the particular region be selected as: *R*_1_ ≔ *τ*_1_ × *τ*_2_ ∈ [0,0.8]×[0,0.8], with Δ*τ*_⋅_ = 0.01, i.e. *N* = 80, and *ε* = 10^−6^. The linear interpolation formula, according to Eq (16), is used. Entries of the found $\hat{\bar{Τ}}$ values are depicted in Fig 3 and compared with the stable/unstable region calculated by the QPmR of a rough delay resolution of Δ*τ*_⋅_ = 0.01. Supporting files S2A, S2B and S2C Table display signs of the rightmost CQP roots from the QPmR, computed leading roots of the ACP along the stability border lines (with the use of pre-warping), and particular values of $\hat{\bar{Τ}}$ and $\hat{\bar{\Sigma }}$, respectively.

**Fig 4:**
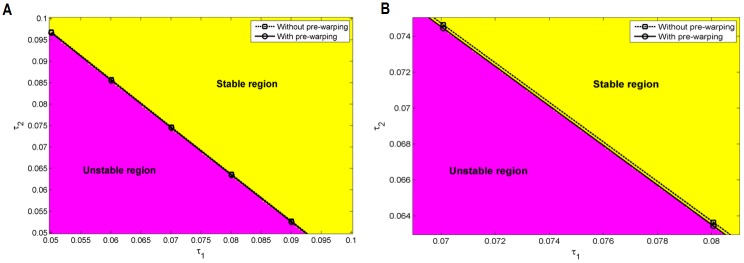
Details of Fig 3 in selected regions. Two regions were selected, a moderately detailed one *R*_2_ (A) and a very detailed one *R*_3_ (B). The stable and unstable areas found by the QPmR for *Δτ*_⋅_ = 0.005 and *Δτ*_⋅_ = 0.001, respectively, are indicated as well.

During computation, we have observed that the convergence of the ACP estimation may be poor when the leading pair of poles found is sufficiently far from the imaginary axis; or, when *α*(**τ**) is near to its extreme (minimum). In such cases, the problem was solved by an interim small change in *k*_*T*_. Both the plots in Fig 3 displaying results with/without the use of pre-warping, are almost indistinguishable by sight; hence, to provide the reader with results in more detail, select subsets *R*_2_ ≔ [0.05,0.1] × [0.05,0.1] and R_3_ ≔ [0.069,0.81] × [0.063,0.075] of *R*_1_ and display found switching delay pairs back-grounded by stable/unstable grid nodes computed by using the QPmR with the gridding Δ*τ*_⋅_ = 0.005 and Δ*τ*_⋅_ = 0.01, respectively, see Fig 4A and 4B.

Table 3 includes the estimated switching delays, the corresponding leading roots found by the QPmR with the precision of 10^−9^, the error with the same meaning as in Table 2 and the *RT* vector within the region *R*_2_.

**Table 3:** Highlighted results of Algorithm 1—Inside region *R*_2_.

Mapping	$\bar{\tau }$	*s*_0_	Error	*RT*
Eq (9)	(5.00643, 9.68377)e-2	-3.578e-4 + 3.847336i	2.03e-4	(-1.980057–3.902248i, -1.776911–3.712380i)
	(6.00732, 8.57107)e-2	-3.592e-4 + 3.849544i	2.02e-4	(-1.979616–3.901380i, -1.784657–3.703965i)
	(7.00747, 7.46430)e-2	-3.604e-4 + 3.851478i	1.99e-4	(-1.979793–3.901079i, -1.793172–3.696423i)
	(8.00698, 6.36342)e-2	-3.609e-4 + 3.853141i	1.98e-4	(-1.980603–3.901336i, -1.802458–3.689744i)
	(9.00595, 5.26837)e-2	-3.611e-4 + 3.854537i	3.47e-4	(-1.982059–3.902144i, -1.812522–3.683919i)
Eq (12)	(5.00665, 9.66366)e-2	-4.9e-6 + 3.848074i	2.4e-6	(-1.977402–3.901955i, -1.774661–3.711958i)
	(6.00741, 8.55114)e-2	-5.5e-6 + 3.850279i	3.0e-6	(-1.976951–3.901107i, -1.782401–3.703575i)
	(7.00743, 7.44455)e-2	-5.6e-6 + 3.852210i	2.1e-6	(-1.977117–3.900824i, -1.790908–3.696065i)
	(8.00682, 6.34384)e-2	-5.1e-4 + 3.853870i	2.7e-6	(-1.977919–3.901097i, -1.800189–3.689416i)
	(9.00568, 5.24897)e-2	-4.3e-4 + 3.855262i	3.3e-6	(-1.979368–3.901922i, -1.810246–3.683621i)

The results of Algorithm 1 within region *R*_2_ = [0.05,0.1] × [0.05,0.1]; Root tendency (*RT*) was defined in Eq (3). The rest of the quantities have already been defined in Table 2.

As can be seen from Table 3, the results correspond to those in Table 2—and, once again, one can state that the use of pre-warping mapping gives two orders higher precision. The overall closeness to the stability bound computed by the QPmR is very good.

**Fig 5:**
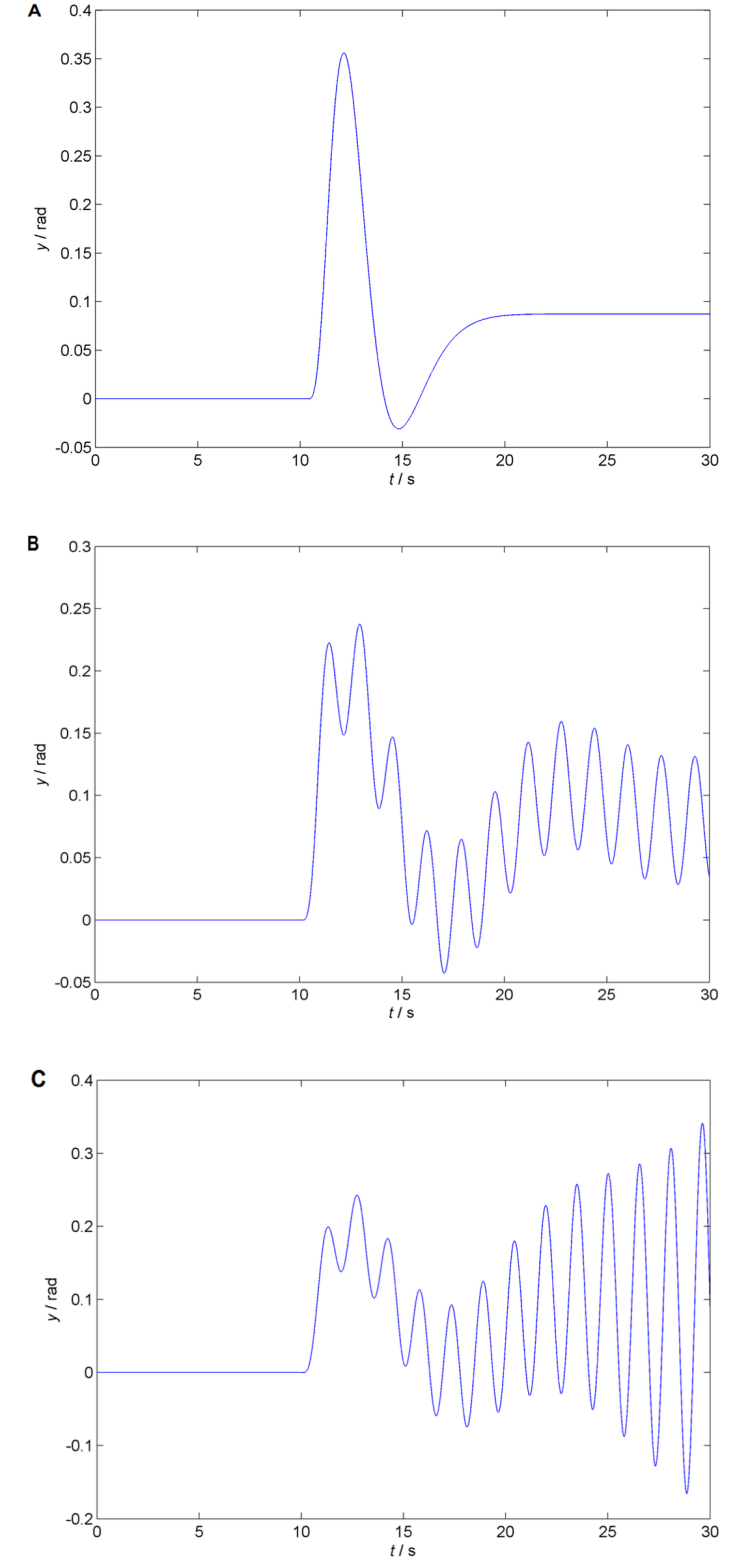
Time domain results verification. The three plots of the system’s step reference time responses for three different delay vectors are displayed. Whereas the nominal setting **τ** = (0.3,0.1) (A) gives a stable response asymptotically tracing the reference signal, the second option **τ** = (0.0700747,0.74643) (B) indicates the stability border by steady oscillations, and the last one: **τ** = (0.04,0.04) (C), gives unstable oscillations with a rising amplitude.

Fig 5A, 5B and 5C display feedback output responses for **τ** = (0.3,0.1), **τ** = (0.0700747,0.74643) and **τ** = (0.04,0.04) respectively, when there is the reference step change Δ*r* = 5° = *π*/36rad from the zero steady state on the control feedback input at time *t* = 10s, to verify the result in the time domain. Apparently, delay values from the region determined as stable (namely, the nominal ones) yield a stable step response (5A); while the ones selected from Table 2 as switching delay result in a steady periodic output of a skater’s position angle deviation (see 5B), and the third chosen delays give unstable response (5C). Let us recall that—amazingly, smaller delay values deteriorate stability in this case. Note that S1 File includes simulation MATLAB^®^ (Simulink) scheme of the control loop.

### Computational complexity

Although it is definitely not a difficult or crucial task in the case of the DDS algorithm introduced here, the (asymptotical) computational complexity should be discussed anyway. Parameterized statements are presented first, followed by practically measured data from the above numerical bio-cybernetic example. The basic general facts about Algorithm 1 are going to be presented, as well as the complexity of the QPmR.

The course of Algorithm 1 can be divided into two parts: a cyclic and a non-cyclic one. The non-cyclic part of the program implementation has the time consumption *ϑ*_*nc*_; consisting of the initialization and the CQP definition. The cyclic one has two subdivisions: with, or without, the RF, *ϑ*_*c*,*nRF*_, *ϑ*_*c*,*RF*_, respectively. The former one mainly includes the iterative polynomial approximation and the leading zero calculation, whereas the latter one expresses time for the successive RF interpolation when the imaginary axis is crossed. The following statement can be formulated:

*Proposition 2*: The computational complexity of Algorithm 1 reads:

$$\begin{array}{l} C\left(N,L\right)=\vartheta_{nc}+{\left(N+1\right)}^{L}\vartheta_{c,nRF}+c_{1}{\left(N+1\right)}^{L-1}\vartheta_{c,RF}=\vartheta_{nc}+\left(c_{2}\left(2L-1\right)+N+1\right){\left(N+1\right)}^{L-1}\vartheta_{c,nRF}\\ c_{1},c_{2}\in R_{+}\backslash \infty \end{array}$$ (24)

The proof of Proposition 2 is given in the Appendix.

As mentioned above, the QPmR can be used in Algorithm 1 instead of steps 6 and 7 to avoid the computation of the ACP; or within the direct procedure, in which for a sufficiently fine grid of discretized delays, the CQP zeros inside a defined region are computed. Regarding the latter case, its complexity can be expressed simply as:

$$C_{QPmR}\left(N_{QPmR},L\right)=\vartheta_{QPmR,nc}+{\left(N_{QPmR}+1\right)}^{L}\vartheta_{QPmR,c}$$ (25)

where *N*_*QPmR*_ stands for the number of delay discretization steps for the QPmR; *ϑ*_*QPmR*,*nc*_ represents the time that the initialization of the QPmR takes outside the cycle, and *ϑ*_*QPmR*,*c*_ means the time period of the QPmR course over a grid node itself. Note that the duration *ϑ*_*QPmR*,*c*_ significantly depends on the selected region for computation of the zeros, its density, and the desired eventual accuracy.

#### Real computation time efficiency

Finally, let us provide the reader with the concise real computation-time requirements of Algorithm 1, compared to the use of the QPmR for direct leading pole computation inside a defined region. The particular variables and symbols were defined in Eqs (24) and (25). Computational tests were performed by means of a laptop equipped with 32b Windows^®^ 7 Professional, running on an Intel^®^ Core^™^2 DuoCPU P8700@2.53GHz, 4GB memory, in the Matlab^®^ 7.11.0.584 (2010b) environment.

The whole course of the final computing test introduced above of Algorithm 1 took 2595 s, which gives the following mean durations of particular operations: *ϑ*_*nc*_ = 0.54s, *ϑ*_*c*,*nRF*_ = 0.36s, *ϑ*_*c*,*RF*_ = 1.64s for found *c*_1_ = 1.75; hence, it can be calculated that *c*_2_ = 2.66.

Now, let us consider the QPmR and two possible search rectangles in the complex plane and their corresponding computed precisions. The first is chosen as a region of the size $\left[10\text{Re}\left\{\left({\hat{s}}_{0,\left(i\right)}-{\hat{s}}_{0,\left(i-1\right)}\right)\right\}\times 10\text{Im}\left\{{\hat{s}}_{0,\left(i\right)}-{\hat{s}}_{0,\left(i-1\right)}\right\}\right]$ with its center in ${\hat{s}}_{0,\left(i\right)}$, which expresses the currently found leading pole estimation (in the *i*th iteration). For such a case, we have measured the mean values *ϑ*_*QPmR*,*nc*_ = 0.66s and *ϑ*_*QPmR*,*nc*_ = 0.62s (for the selected precision of 10^−8^). The second rectangle has the fixed size of [1×1] centered in ${\hat{s}}_{0,\left(i\right)}$; again, for which we have obtained *ϑ*_*QPmR*,*nc*_ = 0.68s and *ϑ*_*QPmR*,*nc*_ = 0.47s (for the reduced precision of 10^−7^). These results imply that the use of the QPmR for seeking the leading pole instead of the ACP roots computing introduced in Algorithm 1 does not yield a shorter computation time. Moreover, the direct trial-and-error use of the QPmR is hardly applicable; for instance, if the latter region is considered with the discretization step of Δ*τ*_⋅_ = 0.001 (for a sufficiently smooth result), the overall computation time would approximately be 3 · 10^5^ s; see Eq (25).

## Conclusions

The formulation of a novel gridding multiple stability switching delay search algorithm and its verification by a numerical bio-cybernetic example of a human being on a remotely controlled swaying bow were the main objectives of this contribution. The proposed DDS algorithm can be fitted into a group of frequency-domain direct methods, based on an effort to find all characteristic roots (poles) located on the stability border, i.e. on the imaginary axis. Within these methods, it is usually only the upper and lower bounds on the imaginary parts of such poles that can be found by the elimination of delays from the characteristic quasipolynomial [28,33,34]. If the delays are commensurate, a procedure can be relatively fast; however, in the general case of non-commensurate delays, a computationally-lengthy iterative reduction procedure ought to be performed [31]. The algorithm presented herein can deal with non-commensurate delays by more effectively omitting a complex mathematical apparatus. The main advantages of the algorithm reside in the fact that it utilizes basic mathematical operations and standard software tools solely; thus, it is simple to implement by engineers and practitioners.

The effectiveness, simplicity, rapidity and accuracy of the gridding algorithm have been tested by the numerical example. The simple control feedback loop with delays system—applied to a skater on a swaying bow, is controlled by a conventional controller. For such a configuration, the marginal stability values of the delay vector were found by the proposed DDS algorithm; these results can be utilized practically, e.g. in biomechanics. The example presented here, proved that the procedure is computationally more effective than the direct use of the QPmR algorithm [43].

The formulation of the Algorithm 1 introduced above, represents its basic version. There are some natural possibilities regarding how to suggest a modification—for instance, the delays′ discretization can be performed with diverse settings $N_{k_{1}}\neq N_{k_{2}}$ for some *k*_1_ ≠ *k*_2_ rather than a constant *N*, or with a non-constant Δ*τ*_*k*,*j*_ for various *k*,*j*. In addition, it is not necessary to take *τ*_*k*,0_ = 0; however, in such a case, a sufficiently accurate estimation of the primal pole ${\hat{s}}_{0,\ldots ,0}$ has to be made, e.g. by means of the QPmR. These topics might be solved in future research. Its applicability is limited to retarded and strongly stable neutral delay systems—which also yields further natural possible research directions. In addition, the numerical example has shown that the crucial step consists in the determination of a suitable sampling period that influences the accuracy and the convergence rate.

## Appendix

*Proof of Theorem 1*: Assume the linear interpolation first. Then, this can be written:

$$z^{-\left(n+m\right)}=z^{-\left(n+1\right)}z^{1-m}$$ (26)

where the left-hand factor has the integer exponent 1 − *m* ∈ (0,1). Let the intention be to find: $a_{0},a_{1}\in \mathbb{C}$ such that *z*^1−*m*^ ≈ *a*_0_ + *a*_1_*z*. Now employ the Taylor series expansion at any *z*_0_:

$$z^{1-m}={\left[z_{}^{1-m}\right]}_{z=z_{0}}+{\left[\left(1-m\right)z^{-m}\right]}_{z=z_{0}}\left(z-z_{0}\right)+c_{1}{\left(z-z_{0}\right)}^{2}$$ (27)

for some $c_{1}\in \mathbb{C}\backslash \infty$ emerging from the remainder. If |*z* − *z*_0_| is sufficiently small, the last term can be neglected. Then, by substituting Eqs (27) into (26) and applying simple algebra on Eq 26 the eventual formula—as in Eq (16) is obtained.

Now consider a proof of Eq (17) and use an analogous idea. Hence:

$$z^{-\left(n+m\right)}=z^{-\left(n+2\right)}z^{2-m}$$ (28)

where 2 − *m* ∈ (1,2). The Taylor series expansion of the latter term can be given as:

$$z^{2-m}={\left[z_{}^{2-m}\right]}_{z=z_{0}}+{\left[\left(2-m\right)z^{1-m}\right]}_{z=z_{0}}\left(z-z_{0}\right)+0.5{\left[\left(2-m\right)\left(1-m\right)z^{-m}\right]}_{z=z_{0}}{\left(z-z_{0}\right)}^{2}+c_{2}{\left(z-z_{0}\right)}^{3}$$ (29)

for some $c_{2}\in \mathbb{C}\backslash \infty$. Again, the cubic term can be cancelled from Eq (29) for some *z* sufficiently close to *z*_0_, and by combining Eqs (29) and (28)—these yield Eq (17), finally.

*Proof of Proposition 2*: The cyclic part must act in every grid node, the overall number of which is (*N* +1)^*L*^. Only if the imaginary axis is crossed by the leading pole, is the *RT* part applied in addition. This happens approximately once or a few times (*c*_1_ < *N*) during the inner-most loop course—and this loop is entered (*N* +1)^*L*−1^ times.

The right-hand side of Eq (24) can be obtained by the supposition that the polynomial approximation is the most time consumptive operation. Whereas, outside the RF, there is only one approximation—the number of 2(*L* − 1) + 1 = 2*L* − 1 such operations inside the RF is performed, i.e. $\vartheta_{c,RF}={\tilde{c}}_{2}\left(2L-1\right)\vartheta_{c,nRF}$ where ${\tilde{c}}_{2}>0$ assumes other low-time consuming operations within the cycle. Substituting this and $c_{2}=c_{1}{\tilde{c}}_{2}$ into the left-hand side of Eq (24), the right-hand value is obtained. Note that for a particular *N*, *L* one can also write *C*(*N*, *L*) = *ϑ*_*nc*_ + *c*_3_(2*L* + *N*)(*N* + 1)^*L*−1^*ϑ*_*c*,*nRF*_, *c*_3_ > 0.

## Supporting information

S1 Table The eventual approximated leading pole loci for Table 1. Leading pole estimations of the ACP received from steps 6 and 7 of Algorithm 1, which gives rise to errors introduced in Table 1.
(XLSX)

S2 Table Results of the numerical experiment according to Algorithm 1. Signs of the rightmost quasipolynomial roots from the QPmR for Fig 3 are given in part A: The data table includes signs of the CQP roots calculated via the QPmR that give rise to the stable and unstable regions in Fig 3. Computed leading roots of the ACP along the stability border line of Fig 3 are provided in part B: The table displays the computed leading roots of the ACP for delay values in the vicinity of the stability border and also their real parts for some other selected delays, as in Fig 3. The pre-warping strategy is used here. Computed switching delays and poles for Fig 3 are introduced in part C: In the table, the reader can find values sets of and for both the strategies (with or without pre-warping) to support data in Fig 3.
(XLSX)

S1 File A simulation scheme of the skater stabilization. This *.mdl file from MATLAB^®^ enables to test the feedback response for various *τ*_1_ and *τ*_2_ that can be set inside the "Skater model" block.
(MDL)

## Abbreviations

ACP: Associated Characteristic Polynomial
CQP: Characteristic Quasipolynomial
DDS: Delay Dependent Stability
grad: Gradient
LMI: Linear matrix inequality
QPmR: QuasiPolynomial mapping Rootfinder
RF: Regula Falsi
*RT*: Root Tendency
sgn: Signum
